# Ligand-free estrogen receptor activity complements IGF1R to induce the proliferation of the MCF-7 breast cancer cells

**DOI:** 10.1186/1471-2407-12-291

**Published:** 2012-07-16

**Authors:** Anne-Marie Gaben, Michèle Sabbah, Gérard Redeuilh, Monique Bedin, Jan Mester

**Affiliations:** 1 Inserm U938, Centre de Recherche Saint-Antoine, Hôpital Saint-Antoine, Bâtiment Kourilsky, 34 rue Crozatier, 75571, Paris cedex 12, France; 2 Université Pierre-et-Marie-Curie Paris 6, 75005, Paris, France

## Abstract

**Background:**

Ligand-dependent activation of the estrogen receptor (ER) as well as of the insulin-like growth factor type 1 (IGF1R) induces the proliferation of luminal breast cancer cells. These two pathways cooperate and are interdependent. We addressed the question of the mechanisms of crosstalk between the ER and IGF1R.

**Methods:**

We evaluated the mitogenic effects of estradiol (E2; agonist ligand of ER) and of insulin (a ligand of IGF1R) in the MCF-7 cells by flow cytometry and by analyzing the cell levels of cell cycle-related proteins (immunoblotting) and mRNA (RT-QPCR). To verify the requirement for the kinase activity of Akt (a downstream target of IGF1R) in the mitogenic action of estradiol, we used shRNA strategy and shRNA-resistant expression vectors.

**Results:**

The activation of the ER by E2 is unable to induce the cell cycle progression when the phosphatidyl inositol-3 kinase (PI3K)/Akt signaling is blocked by a chemical inhibitor (LY 294002) or by shRNA targeting Akt1 and Akt2. shRNA-resistant Akt wild-type constructs efficiently complemented the mitogenic signaling activity of E2 whereas constructs with inactivated kinase function did not. In growth factor-starved cells, the residual PI3K/Akt activity is sufficient to complement the mitogenic action of E2. Conversely, when ER function is blocked by the antiestrogen ICI 182780, IGF1R signaling is intact but does not lead to efficient reinitiation of the cell cycle in quiescent, growth factor-starved MCF-7 cells. The basal transcription-promoting activity of ligand-free ER in growth factor-starved cells is sufficient to complement the mitogenic action of the IGF1R-dependent signaling.

**Conclusions:**

The basal ER activity in the absence of ligand is sufficient to allow efficient mitogenic action of IGF1R agonists and needs to be blocked to prevent the cell cycle progression.

## Background

Therapies based on hormonal manipulations are routinely applied in breast cancer patients whose tumors express estrogen receptor α (ER) (“luminal breast cancer”, some 75–80% of all breast cancers); of these, some 50% benefit from objective responses. The current methods use the inhibition of action of endogenous estrogens by selective estrogen receptor modulators (SERM) such as tamoxifen, or by the suppression of endogenous estrogen production by aromatase inhibitors [1,2].

The primary lack of sensitivity to these therapies of a subset of luminal tumors, as well as the secondary resistance which sets in after an initial response, prevent the cure of patients from their cancer by hormonal therapy alone. There has been extensive speculation concerning the mechanisms of resistance. Activating ER mutations or cyclic AMP-dependent phosphorylation [3] account only for a small fraction of relapses. The majority of relapses of breast cancer under hormone therapy probably results from alternative mitogenic pathways triggered by polypeptide growth factors (HER family and IGF) whose actions are transmitted by membrane receptors [4-6]. These pathways have their own impact on cell survival and proliferation but can also phosphorylate the ER (and/or the appropriate transcriptional co-activators) and reinforce its activity. Laboratory research using breast cancer-derived cell lines produced abundant information concerning mitogenic signaling pathways dependent on estrogens as well as on polypeptide growth factors. However, the data presented by different research groups are sometimes contradictory. In particular, the action of estrogens has been reported to be mediated by direct transcription-promoting activity of the ER [7] or by activation of kinase cascades identical to those triggered by cell surface receptors of polypeptide growth factors [8]. Data obtained in our laboratory [9] argue in favor of the direct transcriptional mechanism, but nonetheless confirm the fact that inhibition of the PI3K/Akt cascade by chemical inhibitors or by shRNA prevents the mitogenic activity of estradiol in the MCF-7 cells. The importance of PI3K activity in the IGF-I-induced mitogenic signaling in the MCF-7 cells has been reported by Dufourny et al. [10]. Similarly, although to a lesser extent, the inhibition of the MEK/ERK pathway reduces the mitogenic activity of estradiol (E2). Conversely, it has been reported that the mitogenic activity of IGF1R is blocked by ICI 182780 [11,12]; this anti-estrogen belongs to the category of selective estrogen receptor down-regulators (SERD) since its presence in the cell culture medium leads to a substantial decrease in the content of ER [13]. These data suggest the importance of crosstalk between the signaling by ER and by growth factor receptors.

In this work we have addressed two questions: first, the requirement of the PI3K activity and in particular of the kinase function of its downstream mediator Akt in the estrogen-induced cell cycle progression, and second, the interplay between the ER- and IGF1R-dependent mitogenic signaling pathways.

## Methods

### Cell culture

Breast cancer-derived cell lines (MCF-7, MELN) were propagated in DMEM supplemented with 10% fetal bovine serum (FBS).

For experiments, the cells were seeded at approximately 20.10^3^/cm^2^, allowed to attach overnight, washed twice and placed in phenol red-free, serum- free DMEM containing or not 10 nM ICI 182780 for various times as indicated. Mitogenic stimulation was carried out by pipetting the reagents directly into the culture medium in the dish to produce final concentrations: 1 μM estradiol (100-fold excess over the antiestrogen) or 1 μM insulin (sufficient to activate the IGF1R), or 10 nM IGF-I. The final concentrations of other drugs used in some experiments were 20 μM for LY 294002 and 10 μg/mL for cycloheximide.

The distribution of cells among the phases of the cell cycle was evaluated by staining with propidium iodide and flow cytometry.

### Expression vectors and shRNA

The shRNA Akt (1 + 2) vector was a gift of Dr. F. Czauderna. It contains a sequence (cloned under pol III promoter in a U6 vector) common to isoforms of Akt1 and Akt2 [14].

The effective and specific suppression of Akt expression by this sequence in the HeLa cells has been verified by these authors and we have confirmed this suppression in the MCF-7 cells (Additional file 1: Figure S1).

To create wild-type Akt1 (Akt1R) and Akt2 (Akt2R) vectors, resistant to shRNA Akt (1 + 2), we used the HA-Akt1 and HA-Akt2 expression vectors (obtained from Xiao GH, Altomare DA and Testa JR, Fox Chase Cancer, Philadelphia, USA) [15]. We introduced silent mutations of 3 codons within the shRNA target common sequence. The following sequences were used: **Akt1**, forward 5′CCAACACCTTCATCATCCggTgTCTCCAgTggACCACTgTCATCg-3′; reverse 5′-CgATgACAgTggTCCACTggAgACACCggATgATgAAggTgTTgg-3′ and **Akt2**, forward 5′-CCAACACCTTTgTCATACggTgTCTCCAgTggACCACAgTCATCG-3′; reverse 5′-CgATgACTgTgTggTCCACTggAgACACCgTATgACAAAggTgTTgg-3′.

To replace the endogenous Akt1 or Akt2 by kinase-dead, sh-RNA-resistant variants, we introduced additional mutation substituting alanine for lysine at position 179 or 181 for Akt1 and Akt2 respectively in the catalytic domains of Akt1R and Akt2R kinases [16]. Point mutation was accomplished by PCR primer mutagens using the QuikChange II Site-Directed Mutagenesis Kit (Stratagene). The following sequences were used: Akt1R/KD (Kinase Dead), forward 5′-CgCTACTACgCCATggCgATCCTCAAgAAgg-3′; reverse 5′-CCTTCTTgAggA TCgCCATggCgTAgTAgCg-3′ and Akt2R/KD (Kinase dead), forward 5′-CgCTAC TACgCCATggCgATCCTgCgAAAgg-3′; reverse 5′-CCTTTCgCAggATCgCCATggCgTAg TAgCg-3′.

Control cells were transfected with the empty pcDNA3 vector. For each transfection, the total quantity of transfected plasmid DNA was completed to 2 μg by the addition of pcDNA3 plasmid (Invitrogen, Life Technologies, Carlsbad, CA). The indicator plasmid used was pCA-Luc (luciferase cDNA cloned downstream of the cyclin A promoter) [17].

### Transfection experiments

Cells were transfected with expression vectors containing: shRNA sequence complementary to Akt1 and Akt2 mRNA (shAkt1 + 2); shRNA-resistant Akt1 or Akt2 (Akt1R and Akt2R); shRNA-kinase dead Akt1 and Akt2 (Akt1R/KD and Akt2R/KD); cyclin A-luciferase (indicator of late G1 phase; β-galactosidase (indicator of transfection efficiency). Transfections were carried out by the Lipofectamine Plus method (Invitrogen) according to the manufacturer’s protocol. After 3 h incubation with the DNA-containing liposomes, the cells were rinsed and incubated 40 h in serum-free, phenol red-free DMEM with 10 nM ICI 182780 prior to stimulation with E2 for additional 24 h. Cells were then lysed in Reporter Lysis Buffer (Promega) and the luciferase and β-galactosidase (Galacto-Star-Applied Biosystems) activities were determined.

### Western blotting

Cells were harvested on ice in a Tris (50 mM, pH 7.4) buffer containing EDTA (20 mM) Nonidet P-40 (0.5%), NaCl (150 mM), dithiothreitol (1 mM), aprotinin (1 mg/mL), leupeptine (1 mg/mL), phenylmethylsulfonyl fluoride (0.3 mM), NaF (1 mM), and sodium orthovanadate (1 mM). The lysates were clarified by centrifugation (10,000 × *g* for 5 min). The total protein concentration was determined by Bio-Rad assay (Bio-Rad, Hercules, CA). 100 μg of total protein were denaturated by boiling in Læmmli buffer containing sodium dodecyl sulfate (1% final concentration) and 2-mercaptoethanol (100 mM) before fractionation by electrophoresis in a polyacrylamide gel (8% or 10% as needed). The proteins were then electrotransferred onto a Hybond membrane and incubated with the appropriate antibodies followed by the peroxidase-tagged secondary antibody. The primary antibodies used were: from Cell Signaling Technology (Beverly, MA) for Akt, phospho-Ser473-Akt, IGF1R (β chain), phospho-GSK3α/β, p21^WAF1/CIP1^, cyclin A; from Santa Cruz Biotechnology (Santa Cruz, CA, USA) for p27 (C-19); from Thermo Fisher Scientific Fremont, (CA, USA) for cyclin D1 (cyclin D1/bcl-1 Ab-1, clone DCS-6); from Millipore Corporation (Temecula, CA, USA) for phospho-ER; from BD Pharmingen (Le Pont de Claix, France) for Rb.

The detection of the signal was carried out with the enhanced chemoluminescence kit (Amersham Biosciences, Saclay, France.).

### mRNA quantification

RNA was isolated by using Trizol (Euromedex). One microgram of total RNA was reverse transcribed with 200 ng random primers (Invitrogen) and ImProm-II reverse transcriptase (Promega) for 60 min at 42°C, in 20 μl final volume.

The cDNA (equivalent of 0.2 μL of the RT reaction mix) was subjected to Q-PCR using Sybr green (Applied Biosciences, Foster City, CA) and appropriate primers. The mRNA contents were evaluated based on the comparative ΔCT method and normalized to the housekeeping gene 36B4 as described previously [18].

## Results

To reduce the risk that experimental results may be influenced by cell heterogeneity, we subcloned MCF-7 cells by limiting dilution. All clones analyzed (18 in total) ceased to proliferate in serum- and estrogen-free medium, and responded to mitogenic stimulation by E2 and insulin. Four clones were further analyzed and found to express the ER and PR (inducible by E2). One of these clones was used in all subsequent experiments.

1. The kinase function of Akt is required for the E2-dependent cell cycle progression.

In our previous work we showed that depletion of Akt1 and 2 prevented the mitogenic signaling by E2 in the MCF-7 cells. At the same time, E2 stimulation failed to induce the activating phosphorylation of Akt on Ser 473. This opened the possibility that Akt may have a function unrelated to its kinase activity, as has been suggested in a different context [16,19]. We therefore produced Akt1 and Akt2 expression vectors carrying silent mutations in the sequence targeted by shRNA, as well as in the kinase domain. As reported by Nakatani et al. [20] and Zinda et al. [21], Akt3 is not expressed in the MCF-7 cells. We tested these constructs for their capacity to “rescue” the mitogenic action of E2 in cells exposed to shRNA targeting Akt1 and 2. The end-point was the activation of the promoter of the cyclin A gene cloned upstream of a luciferase coding sequence, as an indicator of late G1 phase.

**Figure 1:**
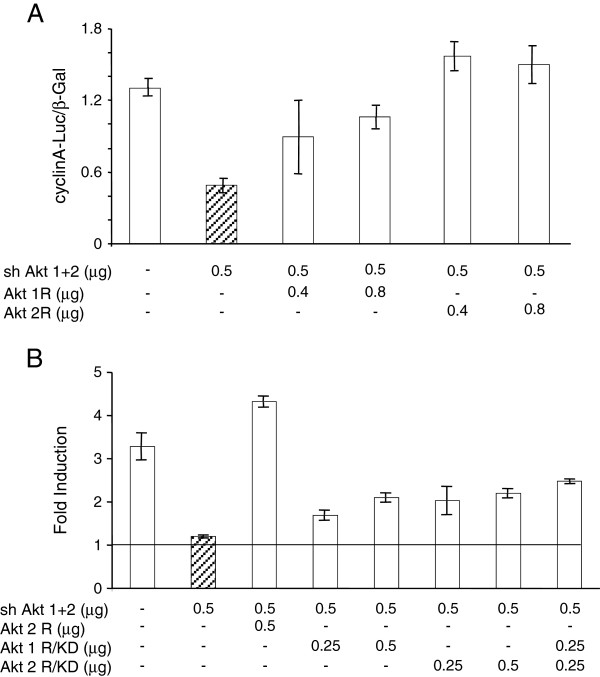
**Akt kinase activity is required for the E2 -induced reinitiation of the cell cycle progression.A**: MCF-7 cells seeded in 35 mm dishes were transfected with shRNA Akt (1 + 2) (0.5 μg per dish) together with expression vectors of shRNA-resistant mutants of Akt1 (Akt1R) or Akt2 (Akt2R) as indicated, and an indicator plasmid encoding luciferase cloned downstream of cyclin A promoter (0.5 μg). A β-galactosidase vector (100 ng) was included to allow the correction for transfection efficiency. For more details see Materials and Methods. The cells were lysed and the activity of luciferase and β-galactosidase were determined. The results shown are means and s.e.m. of triplicates. **B**: The cells were transfected as in A with shAkt (1 + 2), Akt2R, or kinase-dead shRNA-resistant mutants of Akt1 (Akt1R/KD) or Akt2 (Akt2R/KD) as indicated. After transfection, the cells were made quiescent as in A. To a set of dishes of each transfection mix, E2 was added to a final concentration of 1 μM, the remaining dishes were left in the medium containing ICI 182780 (control). The data presented show the induction factor (E2 vs. control) calculated for the luciferase/β-galactosidase activities. The results shown are means +/− s.e.m. of triplicates.

When cells were transfected with the shRNA-expression vector Akt (1 + 2) directed against a sequence shared by Akt1 and 2 mRNAs, the activation of the cyclin A promoter by E2 was blocked and co-transfection of expression vectors coding for shRNA-resistant, wild type kinase variants of the Akt isoforms (Akt1R, Akt2R) restored the cyclin A promoter activation as revealed by the induction of luciferase. Akt2 appeared to be more efficient to restore the full mitogenic effect of E2 than Akt1 (Figure 1A).

Next we compared the wild-type, shRNA-resistant Akt constructs with their kinase-dead counterparts Akt1R/KD and Akt2R/KD. In these experiments, the inclusion of the KD variants resulted in a reduced transfection efficiency documented by the diminished activity of the indicator β-galactosidase. Therefore, we treated groups of dishes with E2 and kept other groups of dishes as controls (in the serum-free medium supplemented with ICI 182780), to calculate the induction factor for the luciferase/β-galactosidase ratios. The results showed that with the kinase-dead mutants, there was only a partial restoration of luciferase induction (Figure 1B) as compared with the wild-type Akt2R used as a positive control. The results of these experiments demonstrate that the kinase function of exogenous Akt is required for efficient rescue of E2-inducible cell cycle progression when endogenous Akt is knocked down.

2. Cells deprived of serum in the absence of ICI 182780 continue to express cell cycle markers.

**Figure 2:**
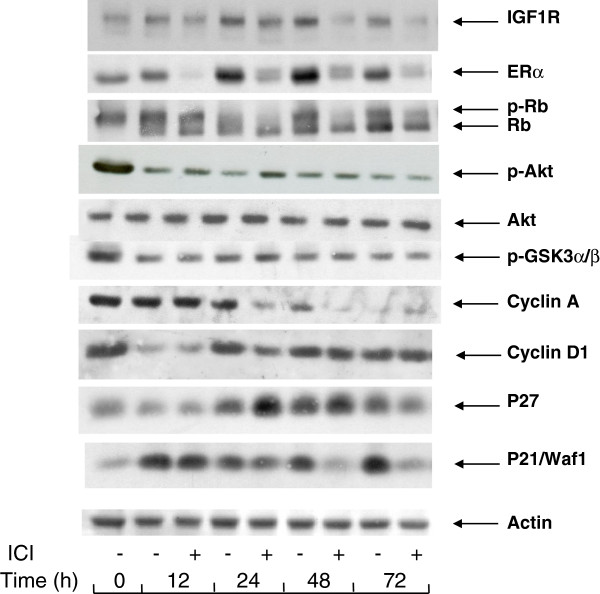
**Changes in the levels of cell cycle-related and signaling proteins during serum- and E2 -starvation.** MCF-7 cells were seeded in 60 mm dishes and allowed to attach overnight. They were then placed in serum- and phenol red-free medium containing or not 10 nM ICI 182780 for periods of time as indicated. After lysis, the different proteins were revealed by Western blotting with the appropriate antibodies.

The arrest of proliferation by depriving the MCF-7 cells of exogenous mitogens was characterized by changes in the cell contents of certain markers of mitogenic signaling of the cell cycle (Figure 2).

Interruption of the mitogenic signaling is illustrated by the changes in the phosphorylation status of the Rb protein, a substrate of cyclin-dependent kinases and a modulator of late G1-phase gene expression. After incubation for 24 h or longer in serum and phenol red-free medium containing ICI 182780, Rb was dephosphorylated, whereas a significant fraction of Rb remained phosphorylated when ICI 182780 was omitted. This indicates that the suppression of ER by the antiestrogen is required for an efficient block of the induction of cyclin-dependent kinases. This conclusion is also supported by the presence of a residual cyclin A in cells deprived of serum in the absence of the antiestrogen whereas in the presence of the antiestrogen, the cyclin A signal is nearly eliminated (Figure 2).

The cdk inhibitory proteins p21^WAF1/CIP1^ and p27 accumulated in cells deprived of serum. Whereas the addition of ICI 182780 in the starvation medium made no difference for p27, it led to a strongly reduced cell content of p21^WAF1/CIP1^ after a transient increase seen at 12 h (Figure 2). The expression of IGF1R also showed a slightly higher level in cells deprived of serum in a medium without the antiestrogen. As the suppression of ER by ICI 182780 leads to a reduced expression of certain genes (see below, section 6), it is likely that the levels of their protein products result from the basal transcription-regulating activity of ligand-free ER.

As expected, in the cells serum-starved in medium with ICI 182780, ER was rapidly eliminated, the signal being near absent at 12 h. In spite of the continued presence of ICI 182780, ER became again detectable at later times. Starvation of serum and E2 in the absence of the antiestrogen led to a progressive accumulation of ER, as seen between 24 and 72 h.

It is to be noted that the cell contents of cyclin D1, a marker of early G1 phase, showed an early decrease at 12 h but then regained about the initial level and remained approximately constant throughout the 72 h incubation in serum-free medium. The presence of ICI 182780 did not reduce the level of cyclin D1 in mitogen-deprived cells (Figure 2).

3. Serum and estrogen deprivation does not eliminate phospho-Akt.

Since the presence of the wild-type form of Akt is a prerequisite for the mitogenic signaling by E2 and since E2 does not induce the activating phosphorylation of Akt, we set out to verify by Western blotting the presence of phospho-Ser473-Akt (p-Akt) in the MCF-7 cells incubated in serum and estrogen-free medium. In these experiments the intensity of the p-Akt signal became weaker during serum deprivation but remained detectable, whether the cells had been incubated in a medium deprived of serum and exogenous estrogens, or in the same medium supplemented with ICI 182780. GSK3α/β a substrate of Akt kinase, showed a similar profile of phosphorylation (Figure 2).

In order to verify that the signal detected with the anti-P-Ser473-Akt antibody represented the phosphorylated Akt rather than a non-specific antigen co-migrating incidentally with Akt, we treated the cell lysates with phosphatase. This treatment abolished the p-Akt signal both in cell lysates prepared from the quiescent MCF-7 cells and in cells treated for 1 h with insulin, a powerful inducer of the PI3K/Akt signaling (see Additional file 2: Figure S2).

The phosphorylation of Akt in the quiescent MCF-7 cells could be a consequence of signaling by an autocrine factor. To test this possibility, we harvested conditioned medium from cells after 48 h of incubation in the absence of serum and we compared the phosphorylation of Akt in quiescent cells placed in fresh DMEM with that detected in cells incubated with the conditioned medium. No difference was seen, suggesting that the Akt phosphorylation resulted from endogenous mechanisms and was not mediated by a secreted autocrine factor (see Additional file 3: Figure S3).

4. IGF1R signal transduction is not sufficient to drive the G1 phase progression.

**Figure 3:**
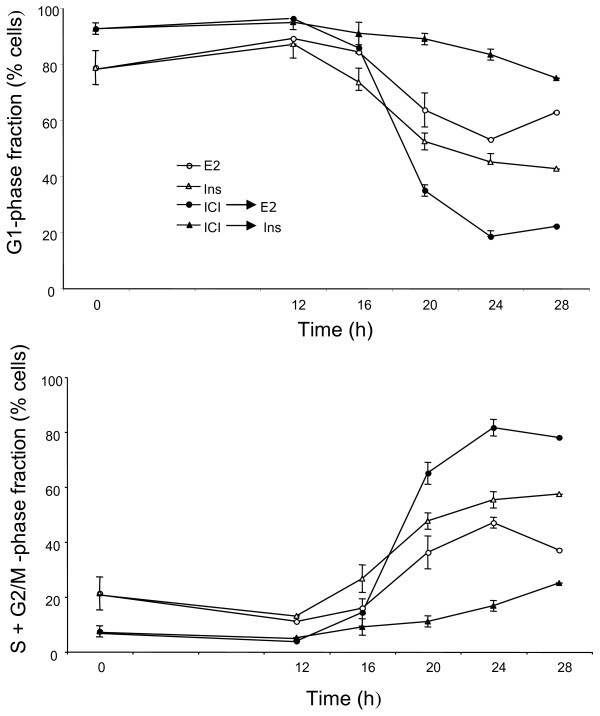
**Reinitiation of the cell cycle progression in quiescent cells.** Blocking the ER function inhibits the insulin-induced reinitiation of the cell cycle progression. MCF-7 cells were placed in serum- and phenol red-free medium containing or not 10 nM ICI 182780 for 48 h. Subsequently the cells were stimulated by addition of insulin (Ins; 1 μM) or E2 (1 μM). The cells were harvested for analysis of their DNA contents by flow cytometry at t = 0, 12, 16, 20, 24 and 28 h as shown. The data (means of 2 to 4 experiments) are plotted as percent of cells in the G1 phase and S phase. S.e.m. are shown (unless smaller than the size of the symbol).

Stimulation of the IGF1R signaling pathway induces a rapid and lasting phosphorylation of Akt. IGF-I and -II, as well as insulin at supra-physiological concentrations, are efficient mitogens in estrogen-deprived MCF-7 cells. Also, simultaneous stimulation of this pathway and of the ER acts in synergy to induce the MCF-7 cells’ proliferation. It has been reported by the laboratory of R. Sutherland that suppression of ER-dependent signaling by ICI 182780 prevents the mitogenic activity of insulin in these cells whereas antiestrogens of the type “SERM” do not show this effect [22]. Varma and Conrad [12] showed that the direct effects of IGF, phosphorylation of IGF1R and of Akt, are unaffected by ICI 182780, in contrast with the inhibition of the mitogenic action. We have addressed the mechanisms underlying the cooperation of the ER and IGF1R pathways. We analyzed the effects of E2 and insulin on the distribution of cells among the phases of the cell division cycle (Figure 3; Additional file 4: Figure S4). Remarkably, even after 48 h incubation in serum-free medium, the MCF-7 cells did not become fully quiescent, with approximately 20% of the total population in S+G2/M-phase (Figure 3, time 0, lower panel). If the serum-free culture medium contained ICI 182780, after 48 h there remained practically no S+G2/M-phase cells. Stimulation with E2 or with insulin triggered the re-entry of G0/G1 arrested cells into the cell division cycle (Figure 3). The most marked mitogenic effect was seen when the cells were fully synchronized by serum-starvation in the presence of ICI 182780 and subsequently stimulated by the addition of E2 (100-fold excess over ICI 182780). In these conditions, insulin produced only a weak and delayed effect. In contrast, insulin was an efficient mitogen when ICI 182780 was omitted from the culture medium (Figure 3, lower panel). 

**Figure 4:**
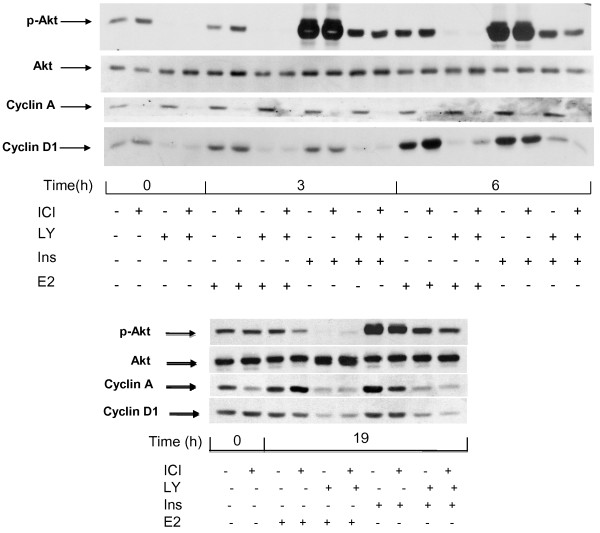
**Effect of the inhibition of PI3K by LY 294002 on the induction of cyclins D1 and A.** MCF-7 cells were starved of serum and E2 during 48 h in the presence or absence of ICI 182780 (10 nM). Last 3 hours of serum deprivation (time −3 h to 0 h), one series of dishes was treated with LY 294002 (LY; 20 μM). Subsequently, at t = 0, all cells were stimulated by the addition of insulin (ins; 1 μM) or E2 (1 μM) for 3 h or 6 h (upper panel) or 19 h (lower panel). After lysis, the different proteins were revealed by after Western blotting using specific antibodies.

These data confirm that pretreatment of the MCF-7 cells with ICI 182780 strongly reduces their sensitivity to the mitogenic action of insulin (Figure 3) while the signal transduction by IGF1R is intact as documented by the strong induction of Akt phosphorylation by insulin in such cells, similar to that seen in cells deprived of serum in the absence of the antiestrogen (Figure 4, upper panel). We also observed an induction of cyclin D1 in cells starved of serum with and without ICI 182780, confirming that this process reflects direct IGFR1 signaling and is not sufficient for the cell cycle progression. There was though a correlation between the induction of cyclin D1 accumulation and the mitogenic action as shown by the FACS data: stronger induction by E2, weaker by insulin in antiestrogen-exposed cells.

The fact that chemical inhibitors of PI3K block the mitogenic signaling in breast cancer cells has been reported earlier [9,23]. This is also illustrated by the effect of LY 294002 on the expression of cyclin A (Figure 4, upper panel). In cells starved of mitogens in a medium without antiestrogen, cyclin A remained detectable, and its content did not diminish during a short (3 to 6 h) incubation with LY 294002 (Figure 4, upper panel). The expression of cyclin A in these conditions is probably the consequence of the incomplete quiescence (Figure 3). When the cells were stimulated with E2 or with insulin for 19 h (late G1 phase), cyclin A was strongly induced and this induction was abolished by LY 294002 (Figure 4, lower panel).

As expected, the effect of IGF-I (10 nM) was the same as that of insulin (1 μM) (Additional file 5: Figure S5).

As ICI 182780 is a SERD-type antiestrogen (inducer of ER degradation), the lack of ER after pretreatment with this compound could be a reason for the diminished sensitivity of the cells to insulin. This is however unlikely to be the case as the reinitiation of the cell cycle progression by E2 in ICI 182780-pretreated cells is actually stronger than that of cells not pretreated with the antiestrogen, in spite of the strong reduction of the cell contents in ER (Figure 2). The recent report of Wardell et al. [24] demonstrates that the efficacy of ICI 182780 as an antiestrogen does not rely on its ability to induce ERα degradation.

5. Effect of the suppression of the PI3K pathway on the expression of cyclin D1 and c-myc protein and mRNA.

We were intrigued by the continuous presence of cyclin D1 in serum- and estrogen-deprived cells, non-suppressible by long-term treatment with ICI 182780. Signaling by the PI3K/Akt pathway favors the accumulation of the cyclin D1 protein by post-transcriptional mechanisms: accelerated translation [25,26] as well as inhibition of degradation of the cyclin D1 protein due to the inhibition of GSK3 α/β through phosphorylation by Akt [27].

**Figure 5:**
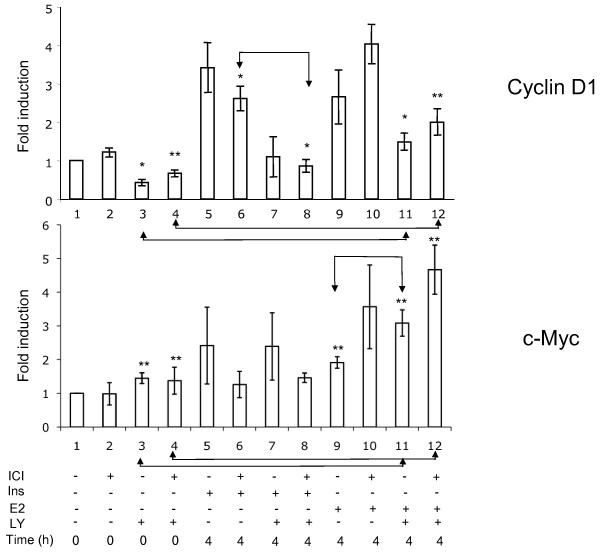
**Effect of LY 294002 on the induction of cyclin D1 and c-myc mRNA.** MCF-7 cells were starved of serum and E2, in the presence or absence of ICI 182780, for 48 h. In one series of dishes, LY294002 was added for the last 3 h of serum deprivation (time −3 h to 0 h). The cells were then stimulated with E2 (1 μM) or insulin (1 μM) for 4 h. The cells were then harvested for the isolation of RNA and RT-QPCR analysis. Fold induction of ΔΔCT (means of three independent experiments performed in triplicate) related to the values at t = 0 in cells starved of serum in the absence of ICI 182780, are presented. The differences between bars 3 *vs* 11, 4 *vs* 12, 6 *vs* 8 and 9 *vs* 11 are significant at p ≤ 0.05 (*) or p ≤ 0.01 **).

In order to verify the role of the basal level of phosphorylated Akt in the expression of cyclin D1, we examined the effect of the PI3K inhibitor LY 294002. A 3 h incubation of serum-deprived cells with this drug strongly reduced the p-Akt signal, indicating that the basal phosphorylation of Akt seen in mitogen-deprived cells depended on PI3K activity. Further, our experiments showed a strong inhibition of the basal cyclin D1 expression by a 3 h exposure of the cells to LY 294002 (Figure 4, upper panel, t = 0). The presence of LY294002 led to a reduction of the contents in cyclin D1 also when the cells were stimulated with either insulin or E2 (Figure 4, upper panel). Next we examined the transcriptional regulation of the *CCND1* gene (Figure 5). The presence of ICI 182780 during serum deprivation did not modify the level of cyclin D1 mRNA. After 48 h in serum-free medium, an incubation for 3 h with 20 μM LY294002 led to a 2 to 3-fold decrease of cyclin D1 mRNA contents, indicating that the basal activity of PI3K was required to maintain the expression of the *CCND1* gene (Figure 5, bars 3, 4 vs. 1, 2). Stimulation of the quiescent cells with either E2 or insulin induced the accumulation of cyclin D1 mRNA (about 3-fold at 4 h). The amplitude of this induction paralleled the pattern of reinitiation of the cell cycle progression (Figure 3): insulin was more efficient when serum deprivation had been carried out without ICI 182780 (Figure 5, bar 5 vs. 6), whereas the effect of E2 was more marked in cells rendered quiescent in the presence of ICI 182780 (bar 9 vs. 10). The induction of cyclin D1 mRNA by E2 was not prevented by LY 294002 (about 3-fold at 4 h compared with the level in control, LY 294002-exposed cells; Figure 5); although the absolute level was lower than that reached without LY 294002, the induction of *CCND1* transcription by estradiol apparently proceeded unhindered (compare bars 3 vs. 11 and 4 vs. 12; differences significant at respectively p ≤0.05 and p ≤ 0.01). On the other hand, the induction of the expression of the *CCND1* gene by insulin was efficiently inhibited by LY294002.

In contrast, in cells cultured in serum-free medium, a 3 h exposure to LY 294002 did not affect the level of the c-myc mRNA (Figure 5, bars 1 vs. 3 and 2 vs. 4). The same result was noted when the cells were stimulated with insulin (absence of effect of LY 294002; bars 5 vs. 7 and 6 vs. 8). The induction of c-myc mRNA accumulation by E2 was actually increased by LY294002 (difference significant for cells maintained without ICI 182780; bars *9* vs. *11*, p ≤ 0.01). It is to be noted that ICI 182780 prevented the induction of c-myc mRNA accumulation by insulin (Figure 5, compare bars 5 vs. 6 and 7 vs. 8).

6. Transcriptional activity of unliganded ER in serum-deprived MCF-7 cells.

**Figure 6:**
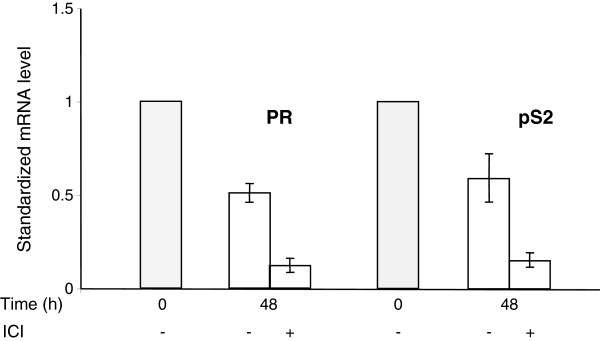
**ICI 182780 is required for the efficient suppression of ER-dependent endogenous transcripts during serum starvation.** Exponentially growing MCF-7 cells (shaded bars) and cells incubated for 48 h in serum- and phenol red-free medium, with or without ICI 182780 (10 nM), were analyzed by RT-QPCR for the ER-inducible pS2 and progesterone receptor (PR) mRNAs. The values of ΔΔCT (means of two independent experiments performed in duplicate) in relation to the exponentially growing cells are presented.

The essential consequence of the presence of ICI 182780 is the suppression of the basal level of ER-dependent gene expression. This was documented by monitoring the levels of two transcripts encoded by genes with estrogen response elements in their promoters, *pS2* and *PR* (progesterone receptor). ICI 182780 caused a strong decrease in the expression of these genes (by approximately 90% after 48 h) whereas in the absence of the antiestrogen their mRNA levels decreased respectively by approximately 50% as compared to those observed in the exponential cells (Figure 6).

**Figure 7:**
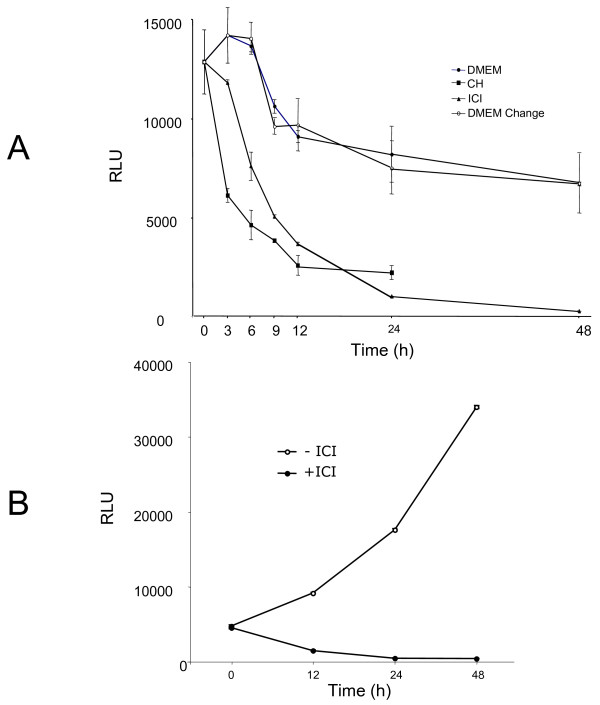
**ICI 182780 is required for the efficient suppression of ER-dependent luciferase during serum starvation of MELN cells.A**. Exponentially growing MELN cells were placed in serum- and phenol red-free medium, with or without ICI 182780 (10 nM) or cycloheximide (CH; 10 μg/mL). In a series of dishes, the medium was refreshed at 3, 6, 9, 12 and 24 h. At the times shown, the cells were harvested for the determination of luciferase activity. The results presented are means of duplicates. **B**. MELN cells were cultivated for more than one month in phenol red-free DMEM supplemented with 5% charcoal-stripped fetal bovine serum and 100 nM insulin. They were then incubated in phenol red-free DMEM without serum and without insulin, with or without ICI 182780 as indicated. At different time intervals, the cells were harvested for the determination of luciferase activity.

In order to obtain a more direct information about the ER-dependent transcription in the absence of ligand, we evaluated the expression of luciferase in the MELN cell line derived from the MCF-7 cells by stable transfection with ERE-TK-LUC [28]. When placed in serum- and phenol red-free medium, the cell content in luciferase varied little, whereas the addition of ICI 182780 led to a rapid extinction of the indicator enzyme, at a rate similar to that caused by the protein synthesis inhibitor cycloheximide, after a delay of about 3 h (Figure 7A). This delay is understandable: cycloheximide blocks all *de novo* synthesis of luciferase protein whereas ICI 182780 prevents the synthesis of mRNA coding for luciferase and not the translation of pre-existing mRNA. To ascertain that the continued expression of luciferase was not due to a possible residual estrogen, we cultured the MELN cells for more than a month (a minimum of 4 passages) in estrogen-free medium supplemented with charcoal-stripped serum plus 100 nM Insulin. The cells were then placed in serum-free medium, without insulin, with or without ICI 182780. Similar results were obtained: ICI 182780 rapidly extinguished the expression of luciferase whereas in the absence of the antiestrogen the level of luciferase increased with time (Figure 7B) 

A possible explanation of these results is the existence of pathways that lead to the phosphorylation of the ER and of co-activators that participate at the regulation of its transcriptional activity. This possibility is sustained by the fact that phospho-Ser118-ER is detected in the serum-deprived MCF-7 cells (data not shown). The mechanism responsible for ER phosphorylation remains unknown at this moment. As in the case of the basal, constitutive phosphorylation of Akt, it is probably the result of an endogenous process, not requiring added or secreted factors.

## Discussion

“Hormone-dependent” breast cancer cells, by definition, require estrogens for their proliferation. Many experimental models used in the literature employ culture conditions where cells (usually MCF-7, a line mimicking luminal breast cancer) are placed in a medium without phenol red (a weak estrogen) and supplemented with FBS treated with active charcoal to remove serum estrogens. However, the dependence of the MCF-7 cells on estrogens is not absolute and, in such estrogen-free media, these cells continue to proliferate, albeit at a slow rate. Charcoal-stripped FBS contains residual polypeptide growth factors (e.g. IGFs), which can stimulate the proliferation of the MCF-7 cells, but even after 48 h incubation in serum-free medium, the MCF-7 cells do not become fully quiescent (Figure 3, time 0). To obtain quiescence, the serum starvation medium needs to be supplemented by a “complete” antiestrogen ICI 182780. Even at quiescence, the cellular phospho-Ser473-Akt (enzymatically active form, dependent on PI3K signaling) is not completely suppressed. We have verified that serum-deprived MCF-7 cells do not secrete autocrine growth factors capable to activate the PI3K/Akt pathway.

We analyzed the mechanisms that may drive the residual cell division cycle in estrogen-deprived cells. We also addressed the question of the role of the PI3K/Akt signal in the crosstalk between ER and IGF1R in the G1-phase progression.

We observed that unliganded ER continues to act as a transcriptional activator in mitogen-deprived cells, and that this action is blocked by ICI 182780. This is documented by our data obtained using the MELN cell line derived from the MCF-7 cells by stable transfection with an ERE-TK-Luc construct [28]. The basal expression of the indicator gene in these cells stabilizes at approximately 50% of the initial level by 48 h and is not eliminated by long-term estrogen deprivation, but is abruptly blocked by the addition of ICI 182780.

The activity of the unliganded ER results also in a higher expression of certain cellular genes as compared with that observed when ER activity is cancelled by ICI 182780. This is the case of the *PS2* gene, which contains an ERE sequence at its promoter, as well as PR (regulated by atypical half-ERE sequences). A higher expression in serum-starved cells without ICI 182780 is also seen for certain cellular proteins not known as ER-targets. For example, p21^WAF1/CIP1^ increases with the time of incubation in serum-free medium when ICI 182780 is omitted. This increase may be an indirect consequence of either the unliganded ER activity during incubation in serum-free medium or of the arrest of the cell cycle (or both). Our laboratory reported earlier that p21^WAF1/CIP1^ cooperates with the ER in the regulation of the expression of genes, apparently with a preference for those genes that are characteristic of differentiation of the mammary gland cells [18].

The cell content of ER is enhanced when the cells are starved of serum and E2 (Figure 2). The expression of ER-target genes in the absence of agonist ligand may be reinforced by this increase during serum starvation [29].

In contrast, the levels of cyclin D1 protein or mRNA were similar irrespective of the presence or not of ICI 182780 during serum deprivation. The *CCND1* gene does not contain ERE, and its induction by E2 relies on the action of ER as a transcriptional co-activator [30]. The sustained expression of *CCND1* in serum and estrogen-deprived MCF-7 cells results apparently from the activity of other transcription factors [31].

Besides its “canonical” role as a Cdki and its cooperation with ER, p21^WAF1/CIP1^ protein appears also to be involved in the activation of Cdk4 [32]. The elevated expression of p21^WAF1/CIP1^ could therefore reinforce the mitogenic signaling resulting from the activation of IGF1R in cells not exposed to ICI 182780.

As we reported earlier, E2 did not rapidly induce Akt phosphorylation [9] (Figure 4) similar observations have been published by others, e.g. [33]. However, the experiments in which we knocked down Akt1 and Akt2 by targeting their shared nucleotide sequence demonstrated that the Akt protein is necessary for the full mitogenic activity of the E2/ER pathway [9]; the present work moreover indicates that the kinase function of Akt is required. Akt2 was more efficient than Akt1, in agreement with the report of Morelli et al. [34]. At the same time, the induction of the PI3K/Akt pathway alone is at best only weakly mitogenic, as illustrated by the weak/delayed effect of insulin on the cell cycle progression in cells where ER activity is suppressed by ICI 182780. Note that overexpression of IGF1R may restore the mitogenic activity of IGF [35]. This is in contrast with the fact that stimulation of the cells with insulin was sufficient not only to ensure the direct actions of IGF1R including the phosphorylating activation of Akt (and of its substrate GSK3β resulting in the post-translational actions such as the stabilisation of the cyclin D1 protein), but also the transcriptional activation of *CCND1*. Our data point to cyclin D1 as the critical element for the estrogen-induced, PI3K/Akt-dependent cell cycle progression. However, cyclin D1 alone is not sufficient to reinitiate the cell cycle progression: cyclin D1 is present in quiescent cells, and, although its level is increased by insulin stimulation (Figures 3 and 5), this is not sufficient for a mitogenic effect [36]. Additional events driven by ER-dependent transcription are necessary. The nature of these additional events is not clear. They do take place in mitogen-deprived cells, albeit at a low rate, due to the transcriptional activity of ligand-free ER and are efficiently blocked by ICI 182780. Activation of IGF1R has been reported to augment the transcription-promoting activity of the ER [37], at least in part via activation of Akt [34]. ER regulates the transcription of numerous genes involved in cellular functions including cell cycle progression, as well as genes coding for other transcriptional regulators, autocrine/paracrine factors, and cell survival [38,39]. It is plausible that the basal expression of such genes is required for triggering the G1-phase progression, in coordination with an enhanced cellular level of cyclin D1. C-Myc is a candidate for this complementary function of ligand-free ER-dependent transcription as it is induced by insulin in cells starved of serum in the absence but not in the presence of ICI 182780 (Figure 5).

Blocking the PI3K/Akt signaling by LY 294002 led to a strong reduction of the *CCND1* transcript, both at quiescence and in mitogen-treated cells. The promoter of the *CCND1* gene contains several regulatory elements on which the PI3K/Akt signal can participate. For instance, transcription of *CCND1* is inhibited by FOXO family transcription factors, which are inactivated by phosphorylation by Akt [40-42] suggesting a mechanism to account for this observation. The effect was selective as, for instance, the expression of the *c-Myc* gene was not reduced.

**Figure 8:**
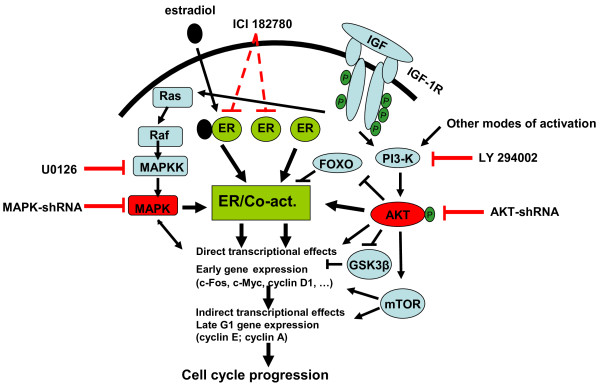
**Mitogenic signaling in MCF-7 cells.** The resumption of cell cycle progression in quiescent MCF-7 cells requires cooperation between two signaling pathways: ER-dependent transcription and PI3K/Akt activity. In the absence of ligand, the ER-dependent transcription proceeds at baseline level. This is sufficient to complement the growth factor-stimulated kinase cascades (Ras/MAPK and PI3K/Akt) to achieve sufficient level of expression of early- and late-G1 genes. The cell concentration of the encoded proteins such as cyclin D1 is also regulated by post-transcriptional processes (e.g. translational regulation by mTOR). The G1/S transition requires in addition the cascade of cyclin-dependent kinases whose activation requires the expression of the appropriate cyclins (D1, E, A) as well as specific phosphorylation and dephosphorylation of the kinases themselves. In the absence of growth factor stimulation, the basal activity of PI3K/Akt is sufficient to complement the full (estrogen-stimulated) transcriptional activation of ER to induce G1-phase progression.

We propose that, in order to induce the cell cycle progression in the MCF-7 cells, both the presence of functional Akt kinase and the transcriptional activation by the ER are required (Figure 8). The basal, ligand-independent transcriptional activation of ER is sufficient to complement the mitogenic signaling via IGF1R/PI3K/Akt; the expression of the *c-Myc* gene may be part of this mechanism. Conversely, the basal level of phospho-Akt present in the serum- and estrogen-deprived cells, with or without ICI 182780, is sufficient to supply the indispensable activity of the Akt kinase needed for the full mitogenic activity of the E2/ER complex. The basal level of phospho-Akt is a consequence of intracellular processes, not requiring added or secreted (autocrine) factors. The precise mechanism which leads to the basal PI3K/Akt activity is not known. The function of the Akt kinase in the mitogenic signaling may be to maintain a sufficient level of phosphorylation of FOXO transcription factors and of GSK3β in order to ensure the transcription of the *CCND1* gene and to stabilize the cyclin D1 protein, necessary for the activation of Cdk4/6 and the primary phosphorylation of Rb. A critical role of cyclin D1 in the breast cancer cell proliferation has been proposed by several laboratories and recently documented in the signaling by anterior gradient-2 [43]. In practical terms, we believe that the development of hormonal therapies based on “full” antiestrogens (lacking agonist action) could improve the outcome of both early and advanced breast cancer. Suppression of estrogen synthesis by the use of aromatase inhibitors is clearly not sufficient to abolish the participation of ligand-free ER in the mitogenic signaling by other growth factors. An additional and substantial improvement would require simultaneous targeting the PI3K/Akt pathway but, until now, no clinically applicable methods have been reported. Also, while most research addressing the need to complement targeted therapies of breast cancer concentrates on the HER family [44], an alternative approach directed at the IGF1R-dependent signaling deserves attention. The interest of the IGF1R pathway is well understood for the development of targeted therapies in other solid tumors including the basal-like, triple negative breast cancer [45]; there is now ample evidence that this pathway is important also in luminal-type breast cancer and may play a role in the recurrence after endocrine therapy. 

## Conclusion

We show that transcriptional activity of the ligand-free estrogen receptor is sufficient to complement the mitogenic action of the IGF1R-induced kinase cascade. Reciprocally, PI3K/Akt activity is required to complement the mitogenic effect of the agonist-activated ER. The basal level of PI3K/Akt present in cells in the absence of exogenous growth factors is sufficient for the full mitogenic effect of estradiol. Thus, both ER and PI3K/Akt need to be targeted for an effective inhibition of the proliferation of hormone-dependent breast cancer cells.

## Abbreviations

cdk: Cyclin dependent kinase; cdki: Cyclin dependent kinase inhibitory protein; E2: Estradiol; ER: Estrogen receptor; ERE: Estrogen response element; GSK: Glycogen synthase kinase; HER: Human epidermal growth factor receptor; IGF: Insulin-like growth factor; IGF1R: Insulin-like growth factor type 1 receptor; PI3K: Phosphatidyl inositol-3 kinase; SERD: Selective estrogen receptor degrader; SERM: Selective estrogen receptor modulator; shRNA: Short hairpin RNA.

## Competing interests

The authors declare that they have no conflicts of interests concerning this work.

## Authors’ contributions

AMG participated at the design, execution and interpretation of the experiments, as well as writing up of the manuscript. MS participated at the RTQPCR experiments and the presentation of the manuscript. MB participated at the cell culture experiments and the presentation of the manuscript. GR participated at the interpretation of the data and the presentation of the manuscript. JM participated at the design, execution and interpretation of the experiments, as well as writing up of the manuscript. All authors read and approved the final manuscript.

## Pre-publication history

The pre-publication history for this paper can be accessed here:

http://www.biomedcentral.com/1471-2407/12/291/prepub

## Supplementary Material

Additional file 1 Figure S1 Knock-down of the Akt signal by shAkt. Transfections were carried out by the Icafectin method (Eurogentec) according to the manufacturer’ protocol with the shRNA as indicated. The cells were serum-starved during 48h and then harvested and analyzed by Western blotting with the Akt antibody. Actin was used as control. (PPT 114 kb) Click here for file

Additional file 2 Figure S2 p- Akt signal is abolished by phosphatase. The cells were starved as in Figure 2 and then stimulated by addition of insulin (1 mM) for 1 h. The cells were lysed in a buffer without EDTA and proteases inhibitors. Portions of lysates (200 μg of total protein) were incubated with calf intestinal alkaline phosphatase (0.05 U/mg protein) for 1 h at 37°C. The lysates were analyzed by Western blotting for Phospho Ser473-Akt. (PPT 114 kb) Click here for file

Additional file 3 Figure S3 Serum-deprived MCF-7 cells do not secrete autocrine factors. The cells were made quiescent in medium with ICI 182780 during 48 h. They were then placed for 6 h in fresh medium (serum- and phenol red-free) with ICI 182780; this was used as conditioned medium (CM). Another series of dishes were stimulated for 1 h or 3 h with CM or with insulin as a positive control, lysed and analyzed for phospho-Ser473 Akt. (PPT 100 kb) Click here for file

Additional file 4 Figure S4 Cell cycle progression of cells stimulated by insulin or E2. MCF-7 cells were deprived of serum in phenol red-free medium with or without ICI 182780 during 48 h, and then stimulated with insulin or with E2 as described in the text. Cells harvested at the different time points were labeled with propidium iodide and analyzed by flow cytometry. The data were evaluated using the ModFit LT software. (PPT 196 kb) Click here for file

Additional file 5 Figure S5 Akt phosphorylation is equally induced by IGF-I and insulin in cells exposed to ICI 182780. Serum- and E2-starved cells exposed or not to ICI 182780 during 48 h were stimulated with IGF-I (10 nM) or insulin (1 mM) for 1 h. The lysates were analyzed for phospho- Ser473 Akt. (PPT 297 kb) Click here for file
