# Supplementary material for: Ligand-free estrogen receptor activity complements IGF1R to induce the proliferation of the MCF-7 breast cancer cells
Source: BMC Cancer. 2012 Jul 16;12:291. doi: 10.1186/1471-2407-12-291 (PMC3476977; doi:10.1186/1471-2407-12-291)
Supplement: Additional file 1 Figure S1 — Knock-down of the Akt signal by shAkt. Transfections were carried out by the Icafectin method (Eurogentec) according to the manufacturer’ protocol with the shRNA as indicated. The cells were serum-starved during 48h and then harvested and analyzed by Western blotting with the Akt antibody. Actin was used as control. (PPT 114 kb) [file 1471-2407-12-291-S1.ppt]

## Slide 1
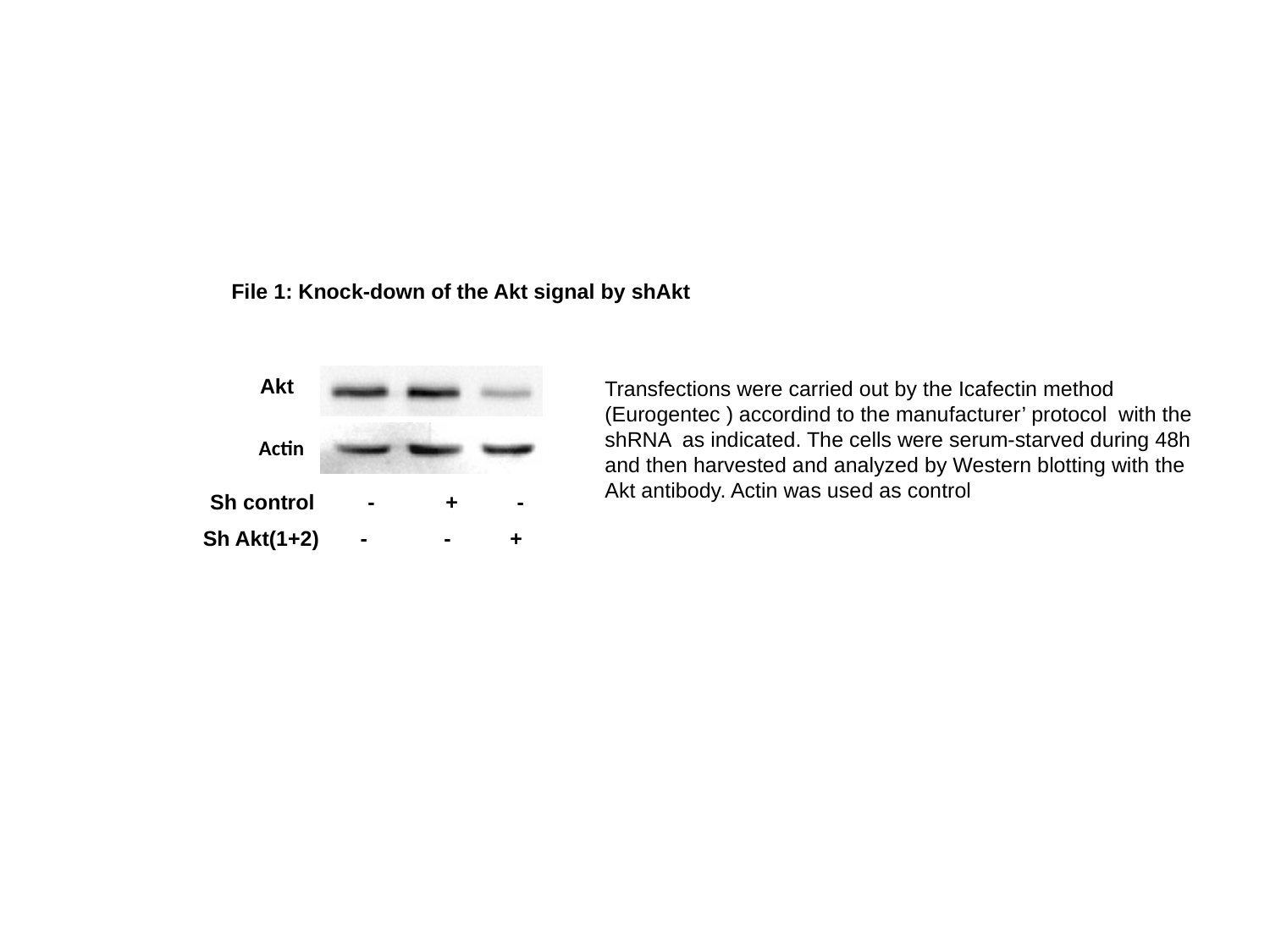

File 1: Knock-down of the Akt signal by shAkt
Akt
Actin
Transfections were carried out by the Icafectin method (Eurogentec ) accordind to the manufacturer’ protocol with the shRNA as indicated. The cells were serum-starved during 48h and then harvested and analyzed by Western blotting with the Akt antibody. Actin was used as control
Sh control - + -
Sh Akt(1+2) - - +
