# Supplementary material for: Ligand-free estrogen receptor activity complements IGF1R to induce the proliferation of the MCF-7 breast cancer cells
Source: BMC Cancer. 2012 Jul 16;12:291. doi: 10.1186/1471-2407-12-291 (PMC3476977; doi:10.1186/1471-2407-12-291)
Supplement: Additional file 2 Figure S2 — p- Akt signal is abolished by phosphatase. The cells were starved as in Figure 2 and then stimulated by addition of insulin (1 mM) for 1 h. The cells were lysed in a buffer without EDTA and proteases inhibitors. Portions of lysates (200 μg of total protein) were incubated with calf intestinal alkaline phosphatase (0.05 U/mg protein) for 1 h at 37°C. The lysates were analyzed by Western blotting for Phospho Ser473-Akt. (PPT 114 kb) [file 1471-2407-12-291-S2.ppt]

## Slide 1
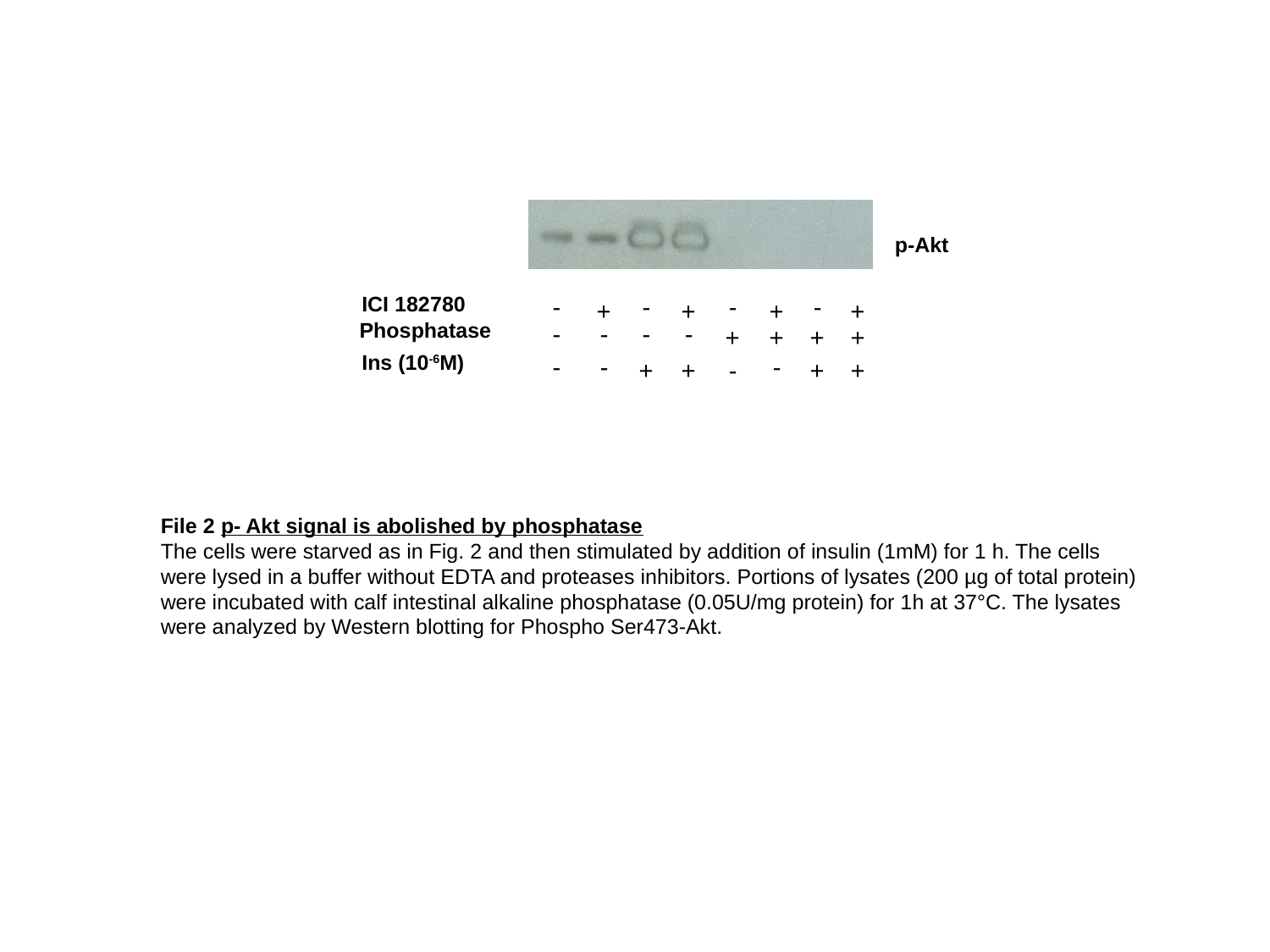

p-Akt
ICI 182780
-
-
-
-
+
+
+
+
Phosphatase
-
-
-
-
+
+
+
+
Ins (10-6M)
-
-
-
+
+
-
+
+
File 2 p- Akt signal is abolished by phosphatase
The cells were starved as in Fig. 2 and then stimulated by addition of insulin (1mM) for 1 h. The cells were lysed in a buffer without EDTA and proteases inhibitors. Portions of lysates (200 µg of total protein) were incubated with calf intestinal alkaline phosphatase (0.05U/mg protein) for 1h at 37°C. The lysates were analyzed by Western blotting for Phospho Ser473-Akt.
