# Supplementary material for: Ligand-free estrogen receptor activity complements IGF1R to induce the proliferation of the MCF-7 breast cancer cells
Source: BMC Cancer. 2012 Jul 16;12:291. doi: 10.1186/1471-2407-12-291 (PMC3476977; doi:10.1186/1471-2407-12-291)
Supplement: Additional file 3 Figure S3 — Serum-deprived MCF-7 cells do not secrete autocrine factors. The cells were made quiescent in medium with ICI 182780 during 48 h. They were then placed for 6 h in fresh medium (serum- and phenol red-free) with ICI 182780; this was used as conditioned medium (CM). Another series of dishes were stimulated for 1 h or 3 h with CM or with insulin as a positive control, lysed and analyzed for phospho-Ser473 Akt. (PPT 100 kb) [file 1471-2407-12-291-S3.ppt]

## Slide 1
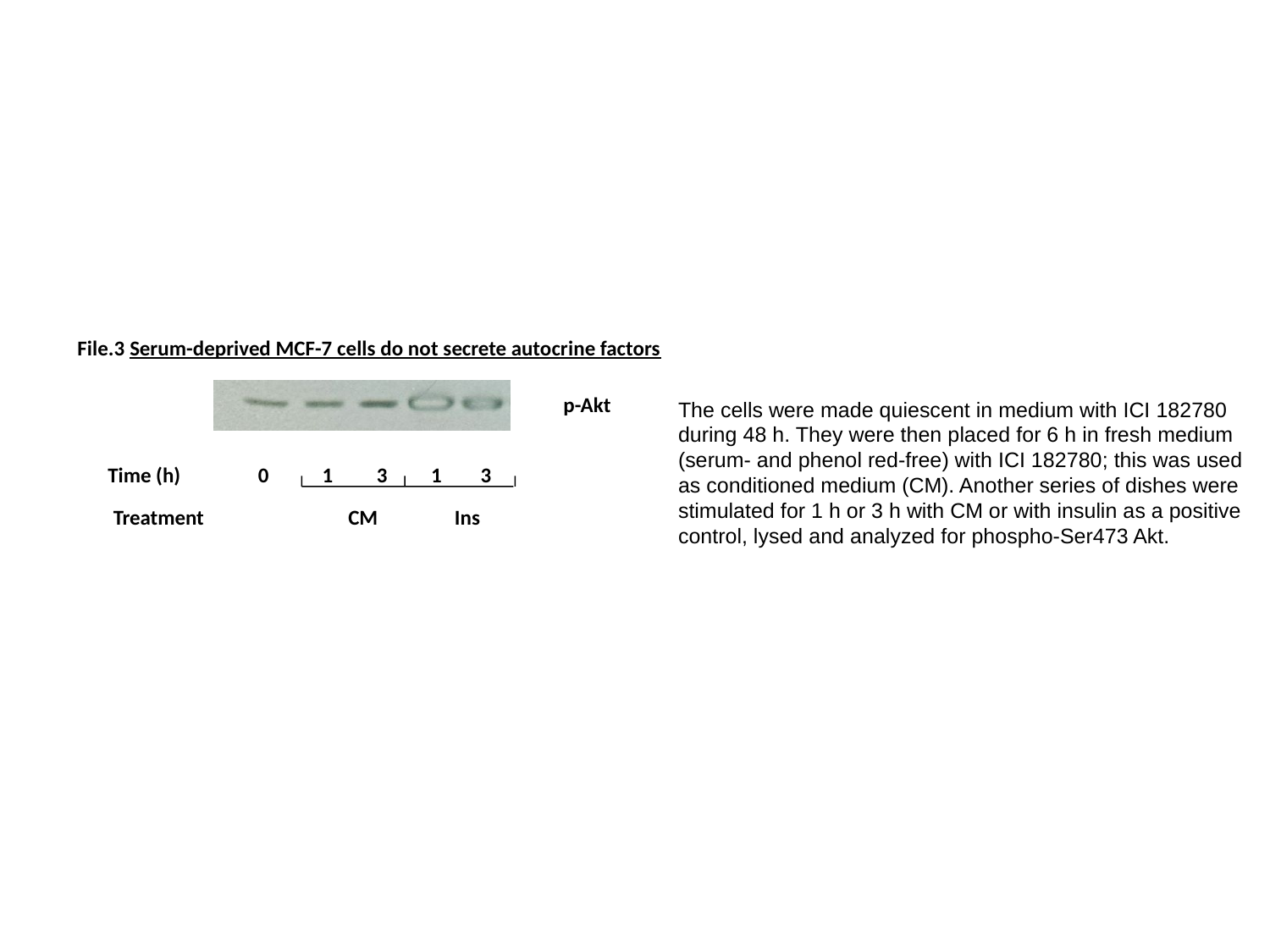

File.3 Serum-deprived MCF-7 cells do not secrete autocrine factors
p-Akt
 Treatment CM Ins
Time (h) 0 1 3 1 3
The cells were made quiescent in medium with ICI 182780
during 48 h. They were then placed for 6 h in fresh medium
(serum- and phenol red-free) with ICI 182780; this was used
as conditioned medium (CM). Another series of dishes were
stimulated for 1 h or 3 h with CM or with insulin as a positive
control, lysed and analyzed for phospho-Ser473 Akt.
