# Supplementary material for: Ligand-free estrogen receptor activity complements IGF1R to induce the proliferation of the MCF-7 breast cancer cells
Source: BMC Cancer. 2012 Jul 16;12:291. doi: 10.1186/1471-2407-12-291 (PMC3476977; doi:10.1186/1471-2407-12-291)
Supplement: Additional file 4 Figure S4 — Cell cycle progression of cells stimulated by insulin or E2. MCF-7 cells were deprived of serum in phenol red-free medium with or without ICI 182780 during 48 h, and then stimulated with insulin or with E2 as described in the text. Cells harvested at the different time points were labeled with propidium iodide and analyzed by flow cytometry. The data were evaluated using the ModFit LT software. (PPT 196 kb) [file 1471-2407-12-291-S4.ppt]

## Slide 1
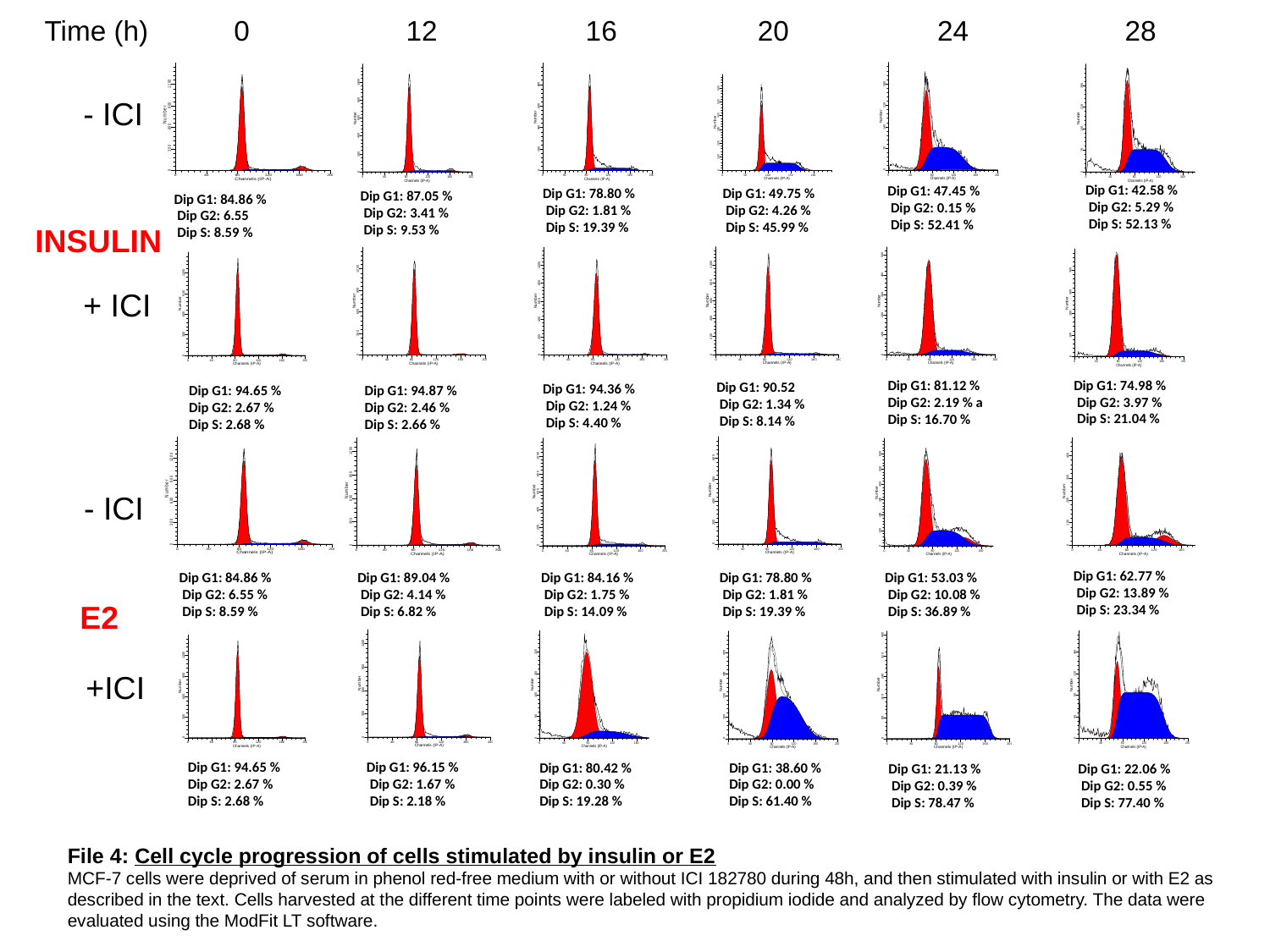

Time (h) 0 12 16 20 24 28
- ICI
Dip G1: 42.58 %
 Dip G2: 5.29 %
 Dip S: 52.13 %
Dip G1: 47.45 %
 Dip G2: 0.15 %
 Dip S: 52.41 %
Dip G1: 49.75 %
 Dip G2: 4.26 %
 Dip S: 45.99 %
Dip G1: 78.80 %
 Dip G2: 1.81 %
 Dip S: 19.39 %
Dip G1: 87.05 %
 Dip G2: 3.41 %
 Dip S: 9.53 %
Dip G1: 84.86 %
 Dip G2: 6.55
 Dip S: 8.59 %
INSULIN
+ ICI
Dip G1: 74.98 %
 Dip G2: 3.97 %
 Dip S: 21.04 %
Dip G1: 81.12 %
Dip G2: 2.19 % a
Dip S: 16.70 %
Dip G1: 90.52
 Dip G2: 1.34 %
 Dip S: 8.14 %
Dip G1: 94.36 %
 Dip G2: 1.24 %
 Dip S: 4.40 %
Dip G1: 94.65 %
Dip G2: 2.67 %
Dip S: 2.68 %
Dip G1: 94.87 %
Dip G2: 2.46 %
Dip S: 2.66 %
- ICI
Dip G1: 62.77 %
 Dip G2: 13.89 %
 Dip S: 23.34 %
Dip G1: 89.04 %
 Dip G2: 4.14 %
 Dip S: 6.82 %
Dip G1: 84.86 %
 Dip G2: 6.55 %
 Dip S: 8.59 %
Dip G1: 84.16 %
 Dip G2: 1.75 %
 Dip S: 14.09 %
Dip G1: 78.80 %
 Dip G2: 1.81 %
 Dip S: 19.39 %
Dip G1: 53.03 %
 Dip G2: 10.08 %
 Dip S: 36.89 %
E2
+ICI
Dip G1: 94.65 %
Dip G2: 2.67 %
Dip S: 2.68 %
Dip G1: 96.15 %
 Dip G2: 1.67 %
 Dip S: 2.18 %
 Dip G1: 80.42 %
 Dip G2: 0.30 %
 Dip S: 19.28 %
 Dip G1: 38.60 %
 Dip G2: 0.00 %
 Dip S: 61.40 %
Dip G1: 21.13 %
 Dip G2: 0.39 %
 Dip S: 78.47 %
Dip G1: 22.06 %
 Dip G2: 0.55 %
 Dip S: 77.40 %
File 4: Cell cycle progression of cells stimulated by insulin or E2
MCF-7 cells were deprived of serum in phenol red-free medium with or without ICI 182780 during 48h, and then stimulated with insulin or with E2 as described in the text. Cells harvested at the different time points were labeled with propidium iodide and analyzed by flow cytometry. The data were evaluated using the ModFit LT software.
