# Supplementary material for: Ligand-free estrogen receptor activity complements IGF1R to induce the proliferation of the MCF-7 breast cancer cells
Source: BMC Cancer. 2012 Jul 16;12:291. doi: 10.1186/1471-2407-12-291 (PMC3476977; doi:10.1186/1471-2407-12-291)
Supplement: Additional file 5 Figure S5 — Akt phosphorylation is equally induced by IGF-I and insulin in cells exposed to ICI 182780. Serum- and E2-starved cells exposed or not to ICI 182780 during 48 h were stimulated with IGF-I (10 nM) or insulin (1 mM) for 1 h. The lysates were analyzed for phospho- Ser473 Akt. (PPT 297 kb) [file 1471-2407-12-291-S5.ppt]

## Slide 1
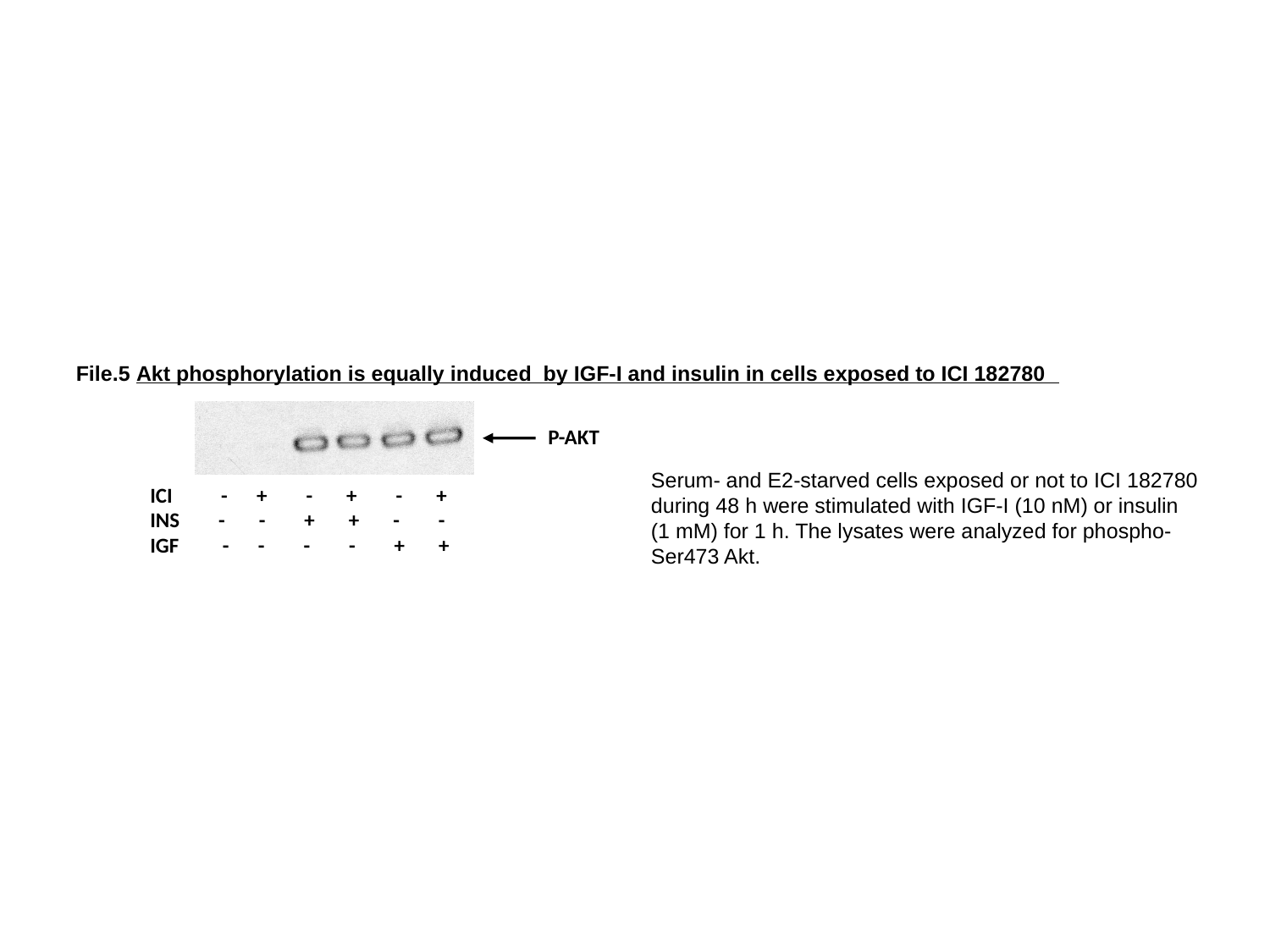

File.5 Akt phosphorylation is equally induced by IGF-I and insulin in cells exposed to ICI 182780
P-AKT
Serum- and E2-starved cells exposed or not to ICI 182780
during 48 h were stimulated with IGF-I (10 nM) or insulin
(1 mM) for 1 h. The lysates were analyzed for phospho-
Ser473 Akt.
ICI - + - + - +
INS - - + + - -
IGF - - - - + +
